# Facilitating a complex behaviour-change intervention: healthcare professionals’ accounts of their journeys to competence and confidence

**DOI:** 10.1186/s12913-025-13676-8

**Published:** 2026-01-12

**Authors:** Ruth I. Hart, Christina Sheehan, Debbie Brewin, Kieran Ayling, Kavita Vedhara, Fran Game, Julia Lawton

**Affiliations:** 1 Usher Institute, University of Edinburgh, Usher Building, 5-7 Little France Road, Edinburgh BioQuarter ‒ Gate 3, Edinburgh, EH16 4UX UK; 2 Lifespan and Population Health, School of Medicine, University of Nottingham, Applied Health Research Building, University Park, Nottingham, NG7 2RD UK; 3 Department of Psychological Medicine, Institute of Psychiatry, Psychology & Neuroscience, King’s College London, 16 De Crespigny Park, London, SE5 8AB UK; 4 Centre for Academic Primary Care, School of Medicine, University of Nottingham, Applied Health Research Building, University Park, Nottingham, NG7 2RD UK; 5 School of Psychology, Cardiff University, Tower Building, Cardiff (Caerdydd), CF10 3AT UK; 6 University Hospitals of Derby and Burton NHS Foundation Trust, Uttoxeter Road, Derby, DE22 3NE UK

**Keywords:** Qualitative research, Behaviour change, Complex intervention, Facilitation, Training, Learning, Reflective practice, Supervision, Intervention delivery.

## Abstract

**Background:**

Interest is growing in whether healthcare professionals from a range of backgrounds can deliver complex behaviour-change interventions effectively. Thus, as part of a wider evaluation of ‘REDUCE,’ a novel, person-centred, cognitive behavioural intervention targeting the self-care behaviours of individuals with a history of diabetic foot ulcers, we explored whether, how, and why, a diverse group of healthcare professionals developed a sense of competence and confidence as facilitators of behaviour change. Our aim was to generate insights to support the recruitment, training, development and retention of appropriately skilled personnel for this and similar behaviour change-oriented interventions going forward.

**Methods:**

We interviewed 15 healthcare professionals who had been appointed and trained to deliver the REDUCE intervention in the context of a randomised controlled trial. We analysed the resulting data thematically.

**Results:**

Our interviewees described diverse backgrounds, routes into the programme, and motivations, and similarly variegated journeys towards competence and confidence as facilitators. They observed how training provided a solid foundation on which to build, but that subsequent learning – substantially self-directed – also played an important part in their development. Interviewees emphasised the particular contributions of experiential learning and reflective practice, noting the key roles that supervision and group support played in the latter, and highlighting how such arrangements helped them to learn from, rather than be derailed by, challenging cases and interactions. Finally, interviewees talked of the returns and rewards of engaging with the REDUCE programme, and how they had benefited both professionally and personally from investing in their own development as facilitators of behaviour change.

**Conclusions:**

Healthcare professionals involved with this behaviour-change intervention were not passive recipients of training and support. Instead, they were self-directed learners who invested actively in their own development. To enable facilitators to reach their full potential, their agency needs to be recognised and interventions organised in ways which enable them to access appropriate experience and support.

**Clinical trial number:**

Not applicable (the manuscript reports on a qualitative study).

**Supplementary Information:**

The online version contains supplementary material available at 10.1186/s12913-025-13676-8.

## Background

High-quality health-related information has been described as ‘the lifeblood of good health and wellbeing … allow(ing) us to understand how to improve our own and our family’s health’[1 (p.7)]. Access to such information is now firmly embedded in UK health policy [1, 2] and its provision is a key component of many healthcare professionals’ work. However, it has become increasingly clear that where behaviour change is the goal, information may be necessary, yet not sufficient [3, 4]. To bring about and/or sustain behaviour change, other approaches, techniques and tools, informed by behavioural theory and science may be needed [2, 4–7]. Hence, in recent years, healthcare professionals have been tasked not only with information provision, but also with delivering brief, evidence-based, behaviour-change interventions, as an adjunct to routine practice [4, 8]. In addition, in the absence of sufficient specialists with expertise in the fields of psychology and/or behaviour change, interest has been growing in whether healthcare professionals from other, disparate backgrounds can facilitate the delivery of more extended and ‘complex’ behaviour-change interventions [9–12]. Such interventions target individuals with complicated health needs (e.g., arising from a chronic condition), have multiple components, and incorporate more sophisticated behaviour-change techniques. They are typically delivered over a number of relatively lengthy (e.g., 30 minutes-plus) sessions.

A small but growing body of research has examined different healthcare professionals’ experiences of, and perspectives on, this emerging area of work. The research provides qualified support for the involvement of non-specialists in the delivery of complex behaviour-change interventions, and some useful insights. For instance, a recent systematic review of 17 research studies investigating the motivational interviewing skills of healthcare professionals working in diabetes care concluded that, subsequent to training, some such professionals employed some relevant techniques [13]. However, as Kaczmarek et al. further observed, the techniques those professionals employed tended to be ones which were less challenging to learn and apply, e.g., increased use of open questions and ‘change statements’ (i.e., articulations of the rationale, or motivation for, change) [13]. Jongebloed-Westra et al. similarly concluded that healthcare professionals (specifically podiatrists) could be trained to use *basic* motivational interviewing techniques successfully in interactions with patients [14]. In terms of the factors supportive of healthcare professionals developing and employing more complex skills in facilitating behaviour change, several studies have highlighted the importance of adequate preparatory training, and/or ongoing, ‘in-service’ training, supervision, and support [13, 15–19]. These findings are valuable, but understanding of the circumstances in which healthcare professionals from different disciplinary backgrounds become competent and confident facilitators of behaviour change remains far from complete.

Thus, as part of a wider evaluation of a novel, person-centred, cognitive behavioural intervention, designed to bring about changes in the self-care behaviours of individuals with a history of diabetes and foot ulceration, we set out to examine facilitators’ development more closely. This complex intervention, ‘REDUCE,’ (see Box 1 for more details) was facilitated by a team of registered and accredited healthcare professionals from a range of disciplines (nursing, podiatry, and psychology/talking therapies). Healthcare professionals were employed specifically to deliver the intervention by the study sponsor (University Hospitals of Derby and Burton NHS Foundation Trust). Further information on the facilitator recruitment process and their employment arrangements is provided in the Methods (Participants and recruitment) section, below.

All healthcare professionals appointed as REDUCE facilitators were required to complete intervention-specific training before being assigned any intervention participants. That training covered the therapeutic approach, use of specific behaviour-change techniques, and, for the benefit of those without relevant expertise, diabetes and diabetic foot health. We describe the organisation and content of the REDUCE facilitator training, and how it evolved over time, in detail elsewhere [20]. In brief, all facilitator training was provided virtually (i.e., on-line) over several days, to small groups of healthcare professionals. It involved a mixture of didactic and interactive components, and was designed and delivered by three experienced Cognitive Behavioural Therapy (CBT) therapists, with contributions from research team members. Trainee facilitators were also expected to undertake some homework/self-study between sessions, such as reading the participant and facilitator handbooks. Following training and alongside delivery of the intervention, facilitators were offered regular one-to-one clinical supervision and/or group-based support, with or facilitated by an experienced and accredited CBT supervisor, based at a collaborating UK university.

In this report, we consider whether, how, and why, the healthcare professionals appointed and trained as REDUCE facilitators perceived themselves as having developed competence and confidence in performing their facilitator role. In particular, we explore the influence and impact of training, supervision, and other, previously unrecognised factors (e.g., independent learning) on facilitators’ development. In so doing, our aim was to generate insights that might support the judicious recruitment, training, development, and retention of appropriately skilled facilitators to REDUCE and/or other, similar behaviour change-oriented programmes in the future.

**Box 1:** Outline of the intervention

**REDUCE**: • Is a complex behaviour-change intervention designed for people (≥ 18 years) with diabetes who have recently had a diabetic foot ulcer (DFU) but on enrolment are ulcer-free. • Aims to increase the time people spend ulcer-free, by avoiding or delaying reoccurrence of a DFU, and decreasing time to healing if/when a new DFU develops. • Seeks to establish a suite of behaviours supportive of the above aims, including: effective daily foot-checking; rapid help-seeking (where changes are identified); regular and graded (i.e., appropriate) physical activity; and, personalised strategies for managing low mood.
• Uses an approach informed by Cognitive Behavioural Therapy (CBT), specifically the idea that thoughts, feelings, and actions/behaviours are linked. Modifying thoughts/beliefs may therefore be necessary to change behaviours, and changing thoughts/beliefs *or* behaviours may change feelings. Facilitators work with participants to identify problems (e.g., barriers to behavioural change) and break these down into separate, smaller and more manageable parts for resolution. • Employs a range of recognised behaviour-change techniques including: information provision; goal setting; action-planning; self-monitoring; habit formation; use of prompts; positive re-framing; behavioural experiments; and, behavioural activation. • Is delivered remotely (by telephone or video-call) to participants around England over eight 60-minute sessions. It is supplemented by paper and digital resources available to participants during and beyond those eight sessions.

## Methods

We outline our methods (and our findings), broadly in accordance with the consolidated criteria for reporting qualitative studies (COREQ) [21].

### Design and context

We report here on a qualitative study involving interviews with healthcare professionals appointed and trained to deliver the REDUCE intervention in one or both of two linked (pilot and main) randomised controlled trials (RCTs) (https://www.isrctn.com/ISRCTN15460422 and https://www.isrctn.com/ISRCTN15570706). The qualitative study was underpinned by a pragmatic, ‘common-sense’ critical realism. Maxwell describes this as combining the ontological assumption that an external, physical and social reality exists, independent of our own and our interviewees’ knowledge of it, with a constructivist epistemological position holding that understandings of and perspectives on experience are socially-mediated, situational, and incomplete [22]. Methodologically, our work was informed by the principles and goals of qualitative description – characterised by Sandelowski as a pragmatic, naturalistic approach focused on the production of minimally-theorised descriptive findings, with clear practical application [23, 24]. Ethical approval for both the trials and the nested qualitative work was secured from Wales Research Ethics Committee 3 (references 21/WA/0110 and 22/WA/0053).

### Participants and recruitment

Healthcare professionals were appointed to the REDUCE programme from around the UK, though the trials and intervention were delivered within England. The facilitator role was advertised via the programme’s social media accounts, with snowballing encouraged, and at a major conference attended by healthcare professionals with interests in diabetic foot health. It was described as involving the delivery of a complex intervention, based on a cognitive behavioural model, which seeks to modify the behavioural and psychological risk factors associated with ulcer recurrence and healing. To be eligible for the facilitator role, healthcare professionals needed to have: experience of working at National Health Service (NHS) Band 6–7 or the equivalent; the ability to engage and empower others in behavioural change; and the ability, with training, to deliver the intervention in line with its underpinning cognitive behavioural model. Appointment did not preclude continuing in or taking up other posts, but a minimum commitment to REDUCE of one day (or the equivalent hours) per week was expected. All appointees were employed by the study sponsor and paid for the time involved in: training; delivering REDUCE sessions; attending supervision sessions; and, participating in research activities, including our qualitative interviews.

With regard to this interview study, all facilitators provided written informed consent to take part in an interview at the time of appointment to the REDUCE programme. Towards the end of the pilot and/or main trial, all those who had enrolled in facilitator training were approached by the programme manager to confirm that they would still be willing to be interviewed. After that confirmation was received, their contact information was passed on to the qualitative research team. Their consent was re-confirmed verbally by the interviewer (RIH) before any interviews began.

### Data collection

Interviews were conducted by the first author (RIH), a female, non-clinical researcher with a background in anthropology and applied social research, and more than 20 years’ experience of undertaking qualitative work. RIH had no relationship with participating health professionals prior to her/their involvement with REDUCE. Interviews took place by video-call or phone, as interviewees preferred and technology permitted. Where possible, interviews were scheduled for shortly after healthcare professionals delivered their final REDUCE session and/or as soon as the qualitative research team became aware they had withdrawn from the programme. Most interviews took place between September 2024 and January 2025. They were semi-structured, and supported by a topic guide informed by relevant literatures, the qualitative research team’s prior experience of undertaking interviews with healthcare professionals involved in the delivery of complex health interventions [19], and input from clinical and behavioural science colleagues. See Box 2 for details of the main areas explored in the interviews. The topic guide was employed flexibly, with interviewees encouraged to raise topics and share information they deemed important, and reviewed and refined as data collection and concurrent analysis progressed. Interviews lasted around an hour, were digitally recorded, and transcribed verbatim.

### Data analysis

Interview transcripts were analysed thematically, by two non-clinical qualitative researchers (RIH and JL – the latter being a female, highly experienced qualitative researcher with a background in medical sociology). They used an approach to thematic analysis which was influenced substantially by the work of Braun and Clarke [25, 26]. Both researchers began by immersing themselves in the data, reading the transcripts closely and repeatedly, to get an overview of the data-set and achieve familiarity with its parts, and noting any cross-cutting patterns, discrepant cases, and the thoughts and feelings those things triggered. Then, still working independently, they developed and applied an initial set of codes. Some codes were informed by a priori interests (i.e., were derived directly from the research and interview questions) and might be considered deductive. Others were derived from the data, i.e., inductive, and led ultimately to findings which were unanticipated at the study’s outset. The coding process was recursive and involved continually comparing data with data, data with codes and codes with codes. The latter step facilitated the combining of overlapping codes, and their grouping to form provisional themes. The two researchers then documented their codes and themes in individual analytical reports, which they exchanged and reflected upon. Next, over a series of discussions, the researchers considered: the extent to which their combined themes mirrored and/or were distinct from each other; whether, individually, themes were consistent with, and effectively encapsulated the data; and, the explanatory value of the surviving themes, individually and collectively. Disagreement was minimal and easily resolved through discussion. Once consensus on key themes was reached, RIH returned to the dataset, systematically identifying all relevant material, and collating this using standard MS Office software, so its relationship to the themes could be scrutinised further. Following another round of dialogue, agreement on the parameters and names of the themes was reached. RIH then began drafting an analytical narrative linking the themes together, with relevant illustrations, a refined version of which is presented in the Results section, below. Co-authors, whose professional and disciplinary backgrounds included diabetology, health psychology, CBT and programme management, ‘sense-checked’ the analysis and interpretation of the data.

## Results

We interviewed 15 healthcare professionals. Three were interviewed on two occasions (at the conclusion of both the pilot and main trials). Hence, in total, 18 interviews were conducted. Of our 15 interviewees, 13 had experience of delivering the intervention to patient-participants (with these healthcare professionals being assigned a mean average of 21 participants (range 2–94) and delivering a mean average of 145 intervention sessions (range 9-637)). Our other two interviewees received training, but subsequently chose not to facilitate. We sought to interview a further four healthcare professionals who had similarly embarked on training but did not go on to facilitate. However, those individuals did not respond to communications from the programme manager and/or the interviewer. Our achieved sample is characterised further in Table 1.

**Table 1:** Characteristics of the sample of healthcare professionals (HCPs)

Characteristic	HCPs (*n*)	HCPs (%)
Profession/discipline (current or most recent)		
Podiatry Nursing Psychology/talking therapies	7 6 2	47 40 13
Gender		
Female Male	15 0	100 0
Age on commencing REDUCE training		
30–39 years 40–49 years 50–59 years 60–69 years 70 + years	3 4 3 3 2	20 27 20 20 13
Number of participants (who engaged in ≥ 1 intervention session) assigned to each of the HCPs who facilitated*		
0–9 participants 10–19 participants 20–29 participants 30 + participants	6 0 6 1	46 0 46 8
Number of intervention sessions delivered by each of the HCPs who facilitated* / **		
0–19 sessions 20–39 sessions 40–59 sessions 60–79 sessions 80–99 sessions 100–149 sessions 150–199 sessions 200 + sessions	1 0 4 1 0 3 3 1	8 0 31 8 0 23 23 8

*Participants assigned and intervention sessions delivered in the course of the pilot *and* main trial

**These percentages do not sum to 100 due to rounding

Our analysis generated three intersecting themes: *Different backgrounds*,* routes in*,* and motivations*; *Training and other learning;* and, *Returns and rewards*. Below, we attend to each of these themes, and their associated sub-themes, in turn. Illustrative quotes are provided at relevant points in the text and, additionally, in a **Supplementary File** (which also provides insights into areas of variation in interviewees’ experiences and perspectives). To preserve anonymity, all interviewees have been given a pseudonym.

### Theme 1: different backgrounds, routes in, and motivations

Our interviewees revealed diverse and interesting backgrounds, with the sample including individuals who said they were still working, or had until recently worked, in podiatry, nursing, and/or psychology/talking therapies. Some reported having worked in more than one of these disciplines – for example, starting out as a nurse and then re-training as a podiatrist. A few also had experience in higher education, including delivering pre-registration training. Several were partially retired, and working reduced or part-time hours in one or more other roles.

When asked how they became aware of the REDUCE programme, interviewees recalled a variety of channels. Two had been involved for many years, and delivered an earlier (group-based) version of the intervention. Others explained how they were known to and had been approached by another professional involved with REDUCE (e.g., a research team member and/or a clinical colleague), who, they surmised, perceived them as having the relevant skills and capacity to contribute:

*(Investigator) used to be the consultant in our foot clinic … she knew I’d reduced my hours*,* so she approached me (about REDUCE).* (Cath).

Several described having seen targeted advertising disseminated through relevant professional bodies (e.g., the Royal Colleges) and/or more informal professional networks (see Supplementary File).

These healthcare professionals offered varied, and often multifactorial, motives for applying to be a REDUCE facilitator. The character of the intervention was often cited as a draw, with interviewees highlighting its alignment with longstanding, personal interests in health behaviour and/or psychology (see Supplementary File). Several contrasted the REDUCE model with the more medically-oriented approaches underpinning their routine work and/or indicated frustration with the constraints of practice in the NHS. Such interviewees said they had seized the opportunity to work in more satisfying ways:

*The reason for doing this was … I found when I worked in diabetes*,* is*,* it’s very much a medical model*,* and I got very frustrated with it. And then I got very excited by hearing this. So that’s why I wanted to be involved.* (Josie).

Other interviewees said they were motivated by the prospect of extending their knowledge and/or skills (of preventive footcare or, more commonly, psychological therapies) either to enhance their current practice or to accrue experience supportive of longer-term career ambitions:

*I saw this role*,* and I thought*,* this is brilliant … (because) my kind of goal was to also become a CBT therapist*,* which I’ve now trained as!* (Regina).

Pragmatic factors were also cited by some. For those based in less populous regions, where possibilities for exploring new roles were more limited, remote working was portrayed as offering a welcome, logistically straightforward, development opportunity. A few others noted how they were attracted by REDUCE’s flexible employment arrangements and the rare opportunity (for those employed within the NHS) to work from home:

*(Husband)’s totally retired … (and) I’m 58*,* so I’m slowing down*,* I’m moving to retirement at 60*,* and I wanted to just work at home.* (Frederica).

### Theme 2: training and other learning

#### Training: a lot of new learning

Irrespective of their backgrounds and motives, interviewees’ reflections on the facilitator training were generally positive, with people typically describing this as enjoyable and informative, if demanding and intense:

*It was intense. It was really informative. And it was enjoyable … Yeah*,* I thought the training was good.* (Charlie).

Psychologists, and nurses working in clinical areas other than diabetes, described new learning around risks to diabetic foot health, and the importance – and key pillars of – preventive care:

*There was lots of new things that I learned during the training … how foot ulcers are caused – that was a shock to me … (and) all new to me really – how a person gets a foot ulcer*,* and just how preventable it is.* (Alicia).

Other interviewees, particularly (but not exclusively) those who already knew more about diabetes and/or DFU, highlighted takeaways to do with the CBT-informed approach, and how they could use this to help people conceive and make clinically-relevant changes in their self-care behaviours:

*The main point of training was thinking about … supporting people … empower(ing) them to … manage … their feet themselves*,* and think about what the barriers (to foot-care) might be … (and) helping them … come up with their own solutions … Getting them to think about how they can do things differently.* (Martine).

Though delivered on-line, the training included a significant interactive component: all or some sessions (depending on the training cohort) were delivered live, with questions and comments actively encouraged (*‘You were able to ask questions … it wasn’t a rigid thing*,*’* Winnie), and opportunities provided to role-play the approach (*‘Although I don’t like role play*,* that was quite good*,*’* Cath). Many interviewees reported valuing the chance those activities gave them to work with, and learn from, colleagues from different backgrounds and disciplines, citing the diversity of the cohort as enhancing the already positive training experience:

*We worked with different people … and that was really nice. So … I had another podiatrist … a nurse*,* and a clinical psychologist … she was incredible! There’s just* *so* *much to learn from other people … and different professions. You all have your different lens.* (Susan).

#### Independent learning: addressing perceived gaps in knowledge and skills

Though generally viewing themselves as learning a great deal from the training, interviewees often reported sensing some remaining gaps in their personal knowledge and/or skills. The nature of those perceived gaps varied: for instance, some interviewees with a background in psychology/talking therapies reported feeling a degree of anxiety about the adequacy of their diabetes knowledge (see Supplementary File) and an expectation that they might need to seek support should participants share clinical information with them. Others, from nursing and/or podiatry backgrounds, disclosed concerns about their grasp of the CBT-informed approach.

Several interviewees highlighted their desire to deliver the intervention as well as possible, and deep sense of responsibility to the trial (see Supplementary File). They explained how these concerns had prompted them to engage in additional, independent – and sometimes entirely self-directed – learning. Georgina, for instance, described undertaking various activities to improve her understanding of CBT and associated principles, alongside the facilitator training:

*I watched YouTube videos of CBT sessions*,* and I read a couple of CBT books*,* and I did get myself up to a level where I felt I could be in that group*,* and I could make more meaningful contributions (…) I was behind other people when I started*,* and so I felt I had to catch up.* (Georgina).

Others noted how, once they had started delivering the intervention, they had felt the need to refresh or supplement their knowledge of particular elements (e.g., the ‘Think-Feel-Do’ model and how to communicate that). These interviewees often described having returned to the intervention and/or training resources in preparation for certain sessions or topics (see Supplementary File). Finally, a number of interviewees described delays between finishing training and beginning intervention delivery (typically attributed to labyrinthine employment processes) and a sense of their knowledge, skills and confidence having been eroded in this time period (*‘learning was lost*,*’* Martine). Several recalled how that concern had prompted them to revisit training materials (e.g., slides and videos) and intervention resources (e.g., the REDUCE handbooks) once they were finally cleared to begin work and/or assigned participants.

#### Experiential learning: application, familiarity, and appropriate challenge

Many interviewees, whilst appreciative of the training, also indicated that to become confident and competent facilitators, they needed to put their learning into practice, by working with real participants:

*You can do this role-play training till you’re blue in the face*,* but it’s not until you actually experience it*,* for real*,* that you really learn.* (Frederica).

Timely application of learning was widely viewed as pivotal to capitalising on training, with those who were able to accrue experience quickly citing this as having been immensely helpful:

*I had my first participant in April. So*,* quite soon after I’d finished the training*,* which was good (…) The other lucky thing was that*,* because I had the availability over three days*,* and because there was a lack of HCPs at the time*,* I was asked to take on quite a number of participants*,* quite early on. So … that enabled me to build up that experience quite quickly.* (Georgina).

Unfortunately, not all interviewees had such a smooth transition from training to delivery, or ultimately felt they gained sufficient experience to achieve a real sense of mastery (see Supplementary File).

Reflecting on the ways in which experience supported their development, interviewees highlighted the value of becoming familiar with different aspects of the intervention. Edwina explained how, as the central model became rooted in her mind, she became more comfortable and confident linking participants’ thoughts, feelings and behaviours – something she said she had initially found very difficult:

*The more time you have (the model) in your head*,* the more … (you) just use it in a more … natural (way) … What I’ve always struggled with*,* with a lot of participants*,* has been explaining that model*,* and*,* and seeing that … blank*,* like*,* I don’t know what you’re talking about*,* type-thing … (Now I) pick up on something … quite naturally*,* and say*,* Oh*,* it’s interesting that you were thinking that – how did that make you feel? And*,* what*,* you know*,* what did you do about it?* (Edwina).

Frederica similarly observed how, as she became more familiar with the material, and how to talk about it, she started to feel more at ease. This, she explained, both enhanced her own experience and improved her delivery of the intervention:

*(As) I got more familiar with the material*,* I got more familiar with how to do it. I wasn’t so nervous. I relaxed into it. And it ended up being a conversation*,* rather than being an interrogation … not that I ever made it an interrogation*,* but I did try and step back a bit more*,* and let them talk. So*,* I think I changed. And … grew in confidence.* (Frederica).

Interviewees also talked of the importance of experiencing a level of challenge commensurate with their level of experience of delivering the intervention. Several expressed relief and gratitude to have initially been assigned *‘nice*,*’* engaged people (*‘you know*,* the ones that are really easy to talk to*,*’* Janice), observing that this had helped them get over their initial anxieties and settle into the role. In contrast, working with more reserved or less engaged participants could fuel those anxieties (discussed further below). Interestingly, however, a few more experienced facilitators suggested that at the right time, working with harder-to-engage or otherwise more complex individuals could be a valuable source of learning. Edwina, for example, talked of coming to accept that, as independent adults, participants would form their own views on the utility of the REDUCE messages, and make choices accordingly. Georgina similarly described how working with both those who engaged and those who did not had been key to her learning to *‘meet people where they are*,*’* though the early stages of this process had been uncomfortable:

*Overall*,* I think there was a good balance between people who didn’t engage with it*,* and people who absolutely did. And I became more relaxed … about the people who didn’t. Whereas when you start*,* you … worry*,* “Oh no*,* that was a very short session*,* we didn’t fill the hour*,* he hadn’t filled in this*,* he hadn’t done that” … it did become easier over time.* (Georgina).

#### Reflective practice: transforming challenges into learning opportunities

Interviewees described how they would reflect on sessions, or specific exchanges within those, to inform and improve their practice going forward. Facilitators talked both of reflective activities they had undertaken independently and those they had engaged in with others. With regard to the former, interviewees described ‘work’ they had done in-the-moment and retrospectively. In-the-moment work had involved being aware of habitual practices or tendencies (a common example being directiveness) and making a conscious effort not to default to these. Georgina observed that learning to collaborate, rather than educate, *‘was*,* at times*,* difficult*,*’* and Grace talked of very consciously having to resist the urge to advise, and actively *‘hold myself back.’* Several interviewees also described engaging in deliberate, retrospective scrutiny of their own performance – typically in the early days of delivering the intervention, when levels of self-doubt were high. They recalled reviewing notes on participants and interactions, and, listening back to recordings of their (own) sessions which had been made to enable fidelity assessment. Such interviewees said that any discomfort they felt about being recorded at work was offset by the immense value of being able to review and learn from the recordings themselves:

*The audio-bit was really helpful*,* because I listened back to myself sometimes*,* which was uncomfortable to start with. But actually*,* I learnt from listening back to myself*,* and just hearing how I was coming over.* (Charlie).

Facilitators highlighted how unusual or challenging participants, situations, or exchanges would prompt reflection and sometimes anxiety. They recalled experiences of working with participants who were very reserved, resistant to new ideas, had a multitude of problems, did not consider (foot) health a priority, and/or who disengaged. These sorts of experiences, some suggested, could be very damaging to one’s confidence, when not offset by a body of more positive, rewarding experiences with engaged participants (see Supplementary File). Reflective practice, however, appeared to encourage facilitators to recognise when they were struggling (or operating at the limits of their knowledge and experience) and needed to reach out for support. Interviewees largely talked favourably about their access to and experiences of both individual and group supervision, emphasising the reassurance and practical help those arrangements provided:

*My anxiety was through the roof. I was disproportionately concerned … I felt out of my depth*,* I was scared to get it wrong … I was so anxious about the whole thing … (Supervisor) couldn’t have been more encouraging and reassuring. And she gave me such lovely feedback*,* helpful feedback.* (Susan).

Whilst access to individual, on-demand support from a more experienced therapist helped those with specific concerns or significant issues with confidence, group sessions (facilitated by an experienced and accredited CBT supervisor) were also highly valued by many. Interviewees highlighted learning from and with their peers, noting how whilst participants were unique, there were often commonalities in the challenges they presented. Hence, they could learn from each other’s experiences and perspectives. Interviewees described how interactions with their peers had provided reassurance, made them feel part of a community of learners, and helped them to find ways forward when they were stuck:

*The others … gave me some good pointers to how they might tackle it. Have you tried this … tried that? What was the answer to such-and-such? And really help(ed) me reflect on whether I’d asked the right questions*,* in the right way*,* to bring out … this hard-to-reach person … I found that really helpful.* (Frederica).

Again, the diversity of facilitators’ backgrounds was viewed as enriching the experience, with the different disciplinary lenses group supervision exposed facilitators to generating different perspectives on, and solutions to, thorny problems (see Supplementary File).

### Theme 3: returns and rewards

As well as highlighting the time they had invested in REDUCE, and in equipping themselves to deliver it effectively, many interviewees identified, and indeed emphasised, the substantial rewards they had experienced as a result of involvement. For instance, facilitators consistently commented on how satisfying and/or enjoyable they had found the work they had undertaken in support of the programme. Cath and Charlie talked of the stimulation and sense of achievement they had derived from a new challenge and working (initially) well outside their *‘comfort zone.’* Others highlighted the privilege and pleasure of being able to undertake in-depth, person-centred work, helping participants to make positive changes:

*Having the time with the patients*,* having that one hour*,* is lovely … (Working) as a podiatrist … you’re busy doing things*,* doing a treatment … whereas the REDUCE model was just focusing entirely on the patient*,* listening to their journey*,* and facilitating that … using the resources that were provided. So that was special.* (Janice).

Many surmised that notwithstanding the contexts and constraints in and under which they normally worked, learning from REDUCE had already informed, or would inform, their routine practice. Cath observed how her experiences of REDUCE had already *‘helped me in my role as a Diabetes Nurse Specialist’* and Janice mused *‘it’s consolidated my years of experience*,* it’s kind of pulled everything together.’* Specific areas of transferable learning identified included the importance of clear messaging about pro-active foot-care and rapid help-seeking, with several interviewees in diabetes care saying they had already started having more opportunistic, but in-depth, conversations about foot health (see Supplementary File). Others highlighted how they had begun to adopt a more patient-centred approach, inviting patients to share their concerns and listening more intentionally. Several interviewees noted how REDUCE had enabled them to understand how difficult to implement healthcare professionals’ advice could seem to patients, and the importance therefore of helping people work out how to do the things that really mattered. Alicia explained how this had led to her now exploring patients’ views on any advice she gave, including the barriers they saw to putting it into practice (see Supplementary File).

Some interviewees concluded that REDUCE had fundamentally changed the way they communicated with people, encouraging more active listening, making them more aware of their own thoughts and emotions, and giving them the confidence to engage in difficult or sensitive conversations. Interviewees, including Charlie, viewed this as having had a profound impact on their practice:

*It just helps you talk to people better. And it’s certainly enhanced my clinical work*,* without a doubt. I can have difficult conversations with people that I don’t think I’d be able to have*,* had I not gone through this project*,* because it really helps me break down the parts … (and) become very aware of my own emotions … what’s going on in my own head*,* and whether I’m delivering something based on my emotions*,* rather than on the … patient in front of me … So I think it’s*,* it’s definitely enhanced*,* yeah*,* enhanced my practice.* (Charlie).

Notably, several interviewees saw this ability to embark on and effectively navigate difficult conversations as something that had had valuable impacts in/for their personal as well as professional lives. Josie, for instance, observed how she had learnt to listen more actively, changing both the quality and outcomes of her communications with family and friends:

*The way I communicate with people has changed … I do more active listening … I do that a lot with the kids … It’s not just professionally it’s impacted me*,* I think personally it has as well. So*,* I think … REDUCE has actually helped me as well*,* if I’m honest!* (Josie).

## Discussion

Interest is growing in whether and how healthcare professionals from different disciplinary backgrounds can acquire the knowledge and skills to deliver complex behaviour-change interventions confidently and effectively. Previous research has suggested that this is possible, if healthcare professionals receive appropriate preparation and support [13, 15–17]. Our own findings broadly reinforce that position, revealing how, with training and subsequent supervision, a diverse group of healthcare professionals developed a growing sense of competence and confidence in delivering a novel, person-centred, CBT-informed behaviour-change intervention. Notably, however, our findings also suggest that other, additional development activities may be important; in particular, independent study, and learning from appropriately-challenging experiences, in conjunction with various forms of, and forums for, reflective practice.

As we have documented, the healthcare professionals involved with REDUCE came from a range of disciplinary backgrounds, and had different motives for involvement. An important and unanticipated finding was that this diversity, in particular in professional disciplines, was consistently perceived as a strength, enriching facilitators’ experiences of both their initial training and subsequent group-based supervision. Though a novel finding in this research domain, wider work has similarly reported benefits from inter-disciplinary learning, including mitigation of intra-disciplinary ‘blinders,’ and promotion of critical thinking, creativity, and problem-solving skills [27 (p.4)]. As such, there may be a persuasive case for systematically constructing multi-disciplinary groups for training and supervision in the future, to harness the added-value of professional diversity.

Our findings, however, also suggest some challenges which may arise from disciplinary diversity. Facilitators’ different backgrounds led to different learning needs (e.g., around diabetic foot health and/or the CBT model). These may be harder to meet fully within group-based training. Though the healthcare professionals in our study spoke positively about the initial training they received, they also often reported having undertaken additional, ongoing work to equip themselves for the facilitator role. Indeed, they portrayed themselves as active agents in their own development. In this respect, our interviewees’ accounts bring to mind the andragogical concept of ‘self-directed learning,’ described by Knowles as a cyclical process in which individuals identify their own learning needs, plan how to remedy them, take appropriate action, and evaluate the results [28]. This is a notable observation, as other earlier work has focussed overwhelmingly on what is offered or ‘done to’ healthcare professionals to equip them to deliver interventions [13–15, 18]. In so doing, that work has – inadvertently perhaps – tended to depict healthcare professionals as passive recipients of expert trainers’ knowledge and skills, thereby evoking a model of adult learning which was problematised more than half a century ago [29, 30]. Looking ahead, to support the sort of active, independent learning our interviewees described, and ensure it does not threaten intervention fidelity, there might be value in signposting motivated appointees to approved supplementary resources.

Like Lawton et al. [19], we have highlighted the critical role of experience, documenting how facilitators developed a sense of competence and confidence over time, through application of their training and other learning. In this respect, their accounts bring to mind Kolb’s theory of experiential learning, which conceptualises learning as grounded in experience, and holds action (and reflection – discussed further, below) to be pivotal to ongoing processes of knowledge creation and re-creation [31]. In a distinctive contribution to the practice literature, we have noted the importance of experiencing appropriate challenge, in the sense of working with participants with characteristics and needs commensurate with one’s level of knowledge and skill. Specifically, facilitators’ accounts suggested that experiencing less-challenging interactions was helpful initially, but growth could subsequently come from working with participants with more complex needs and/or who were harder-to-engage. Thus, moving forward, there might be value in assigning participants strategically, allowing new facilitators to build their confidence by working with seemingly more straightforward individuals, before allocating more complex cases to them. Such a triaging process could have benefits for both facilitators and intervention participants, though it is unlikely to be failsafe. This approach would also be consistent with that taken in the NHS Talking Therapies programme (formerly known as Increasing Access to Psychological Therapies), which recommends allocating less complex presentations to new practitioners and, in addition, giving them smaller caseloads to allow time for reflective practice [32].

We ourselves have highlighted how reflective practice, both individually and with the support of a supervisor and/or peers, was portrayed as key to transforming challenges into positive learning opportunities. Again, our findings resonate with to the work of Kolb, which conceives the movement between action and reflection as a driver for learning [31]. We have seen how for these REDUCE facilitators reflection appeared to take a variety of forms, and to occur in different forums and/or contexts. One of these was clinical supervision, the importance and value of which was emphatically confirmed by the experiences of our interviewees. Other authors have, we note, similarly highlighted the value of supervision to healthcare professionals tasked with delivering complex behaviour-change interventions [13, 16, 19]. In addition, however, our interviewees drew attention to diverse benefits they accrued from interacting with their peers in group sessions. Scholars working in the broader field of professional development have similarly described how small groups, such as those constituted as ‘action learning sets,’ can promote the development of communication and problem-solving skills, and facilitate the resolution of specific, work-related problems [33]. Thus, both individual and group supervision are arguably important components of the REDUCE programme, which our findings, in line with those of others [13, 16, 19], suggest should be retained and invested in going forward.

Finally, we have shown how facilitators overwhelmingly viewed themselves as having benefited from involvement with REDUCE. We documented: their sense of satisfaction and enjoyment; perceived impacts on their broader practice; and, wider benefits e.g., in/for their personal, including domestic, lives. Other researchers have similarly described how the acquisition of skills in person-centred and/or CBT-based approaches was viewed by healthcare professionals as having had positive, transformational effects on both their professional practice and personal lives (benefiting their interactions with partners, children, other family members and friends) [34, 35]. Whilst it might be helpful to ensure future candidates are aware of their predecessors’ experiences, and the perceived need of many to supplement training with active, self-directed learning, we would encourage recruiters to balance that out by highlighting the significant and wide-ranging rewards and benefits reported by our interviewees.

### Strengths, limitations and transferability

We explored healthcare professionals’ experiences of developing competence and confidence in facilitating one particular behaviour-change intervention, targeting a specific (though diverse [36]) patient group. Our sample, though relatively small, included all active facilitators, plus two healthcare professionals who embarked on training but chose not to deliver the intervention. Ideally, we would also have spoken to the four further healthcare professionals who did not go on to facilitate, as their perspectives may have offered additional and/or different insights, in particular on recruitment processes and training experiences.

In terms of healthcare professionals’ disciplinary backgrounds, our sample was diverse, giving our findings potential wider relevance. However, it is notable that the REDUCE facilitators were, as a group, relatively experienced. Around half were aged 50 or above, and had commensurate practice experience, sometimes spanning more than one discipline. This is something which readers should take into account when considering the transferability of our findings. Readers should also be aware that individuals signing up to deliver a novel intervention in the context of a trial may be distinctive in other, potentially significant ways. For instance, they may have especially high levels of ‘intrinsic’ motivation with regard to learning and professional development. Were the intervention to be rolled out more widely, it might be necessary to establish conditions that could reinforce intrinsic motivation and/or foster ‘extrinsic’ motivation (e.g., positive experiences and feedback, connectedness with mentors and peers, and financial incentives/rewards) [37]. Nevertheless, future appointees’ levels of motivation for learning/development, and the quality of such activities, may still be fundamentally different.

Moreover, depending on how an intervention is resourced and organised in the future, facilitators could be working in circumstances, or contexts, which present practical barriers (e.g., time or access to technology) to some or all of the forms of active learning our interviewees described. If so, they might struggle to reach the levels of competence and confidence documented here. It is certainly hard to envisage how this sort of work, and investment in learning, could be absorbed within existing workloads, i.e., if appended to routine responsibilities. Dedicated, funded time is required.

As a final observation, whilst we have chosen our words carefully throughout, we recognise that facilitators’ sense of their competence may not reflect objective realities. Fidelity work is underway to assess the extent to which facilitators delivered the intervention as intended: that work will offer important insights complementing those we report here.

## Conclusion

Existing literature has emphasised healthcare professionals’ need for appropriate, high-quality training and support if they are to develop and employ complex skills in facilitating behaviour change. Our findings confirm these inputs are necessary, but suggest that other developmental activities are also important, and that interventions/programmes need to be organised in ways that encourage and enable these. Such activities include opportunities to: undertake independent learning; gain appropriate experience; and, undertake reflection, including in the context of clinical supervision and with peers. These findings have important practical implications for both REDUCE and other future behaviour-change interventions with similar ambitions and staffing models.

**Box 2:** Outline of topics explored in interviews

*Professional background* • Interviewee’s background (e.g., current role, other experience, prior involvement in diabetes education and/or behaviour-change programmes) *Involvement with REDUCE* • How they heard about/got involved with REDUCE • Motives for becoming a REDUCE facilitator • Initial impressions (including how it diverged from routine practice) and expectations *Experiences of the REDUCE training* • When and how this was undertaken • Overall impressions • Views on organisation and delivery • Key content/learning points; novelty of information, ideas and/or approach(es) • Readiness for delivery, anticipated challenges, any concerns or anxieties • Any areas for improvement
*Experiences of delivering REDUCE…* • Transition to intervention delivery • Time period over which delivered, number of participants assigned, intensity of work • What went well and what did not (rewards, challenges, surprises and learning) • Using a collaborative, CBT-informed approach (including the ‘Think-Feel-Do’ model) and recognised behaviour-change techniques (e.g., goal-setting, self-monitoring) • Delivering content relevant to the (4+) target behaviours • Areas of difficulty and/or discomfort (for interviewees); changes in this over time
*…to different participants* • Characteristics of the participants worked with (e.g., age, gender, health, life circumstances, medium used for sessions) • Variability in their responses (to sessions, materials, REDUCE targets, the CBT-informed approach, and the behaviour-change techniques introduced) • Challenging responses, participant characteristics, and/or individuals – and how interviewees coped with these • Adapting to different participants (preparedness, comfort, changes in this over time) *Perceived impact of REDUCE*,* on participants* • If/how target behaviours appeared to change • (Other) ways participants benefited (e.g., other behavioural or psycho-social changes) • Anticipated impact on foot health (and the clinical outcomes measured in the trial) *Resources and support* • Views on the facilitator manual and participant handbook. Any other materials provided, used, or created • Views on and use of the maintenance resources (in digital and/or paper forms) • Administrative and/or technical demands and infrastructure • Session recording and use(s) of audio-files • Experiences of, and views on, individual feedback and supervision • Attendance at, and experiences of, group supervision and peer support • Any unmet support needs; essential and desirable support going forward *Wrapping up* • Feelings about own involvement with REDUCE; professional and/or personal impact(s) • Characteristics of a ‘good’ REDUCE facilitator • Conversations with colleagues, impressions of buy-in to this sort of programme, how to promote it to others (i.e., potential future facilitators) • Opportunities and challenges associated with scaling up the programme (or components) • Any other comments.

## Supplementary Information

Below is the link to the electronic supplementary material.

Supplementary Material 1

## Data Availability

The data drawn on and reported in this manuscript are not publicly available due to risks to individual privacy. However, reasonable requests to access that data will be considered; these should be directed to [j.lawton@ed.ac.uk].

## Abbreviations

CBT: Cognitive Behavioural Therapy
DFU: Diabetic foot ulcer
HCP: Healthcare professional
NHS: National Health Service
