# Supplementary material for: Facilitating a complex behaviour-change intervention: healthcare professionals’ accounts of their journeys to competence and confidence
Source: BMC Health Serv Res. 2026 Jan 12;26:202. doi: 10.1186/s12913-025-13676-8 (PMC12888146; doi:10.1186/s12913-025-13676-8)
Supplement: Supplementary file 1 — Supplementary Material 1 [file 12913_2025_13676_MOESM1_ESM.docx]

**Supplementary File: Additional quotations**

| **THEMES** | **SUB-THEMES AND QUOTES** |
| --- | --- |
| **Different backgrounds, routes in and motivations** | Routes into REDUCE:  *‘2002, roughly … is when I first came into touch with (Investigator) and the team of REDUCE, as it was then … So, I’ve followed this project through … from the beginning (…) (And) the first project … the (group) intervention … I was involved in that, facilitation of that group.’* (Charlie)  *‘I was … training for cognitive behaviour therapy, and I was on … (a) placement … (Supervisor) … said that there was this programme called REDUCE and … to let her know if I wanted to take part.’* (Alicia)  *‘I’m obviously in some podiatry groups on Facebook and I think I saw a flyer or something, of the research trial, and I thought, that’d be really interesting to get involved with.’* (Jamie)  *‘I joined a DSN forum, Facebook forum, involving different DSNs across the UK, and somebody on that forum … advertised (REDUCE).’* (Grace)  Motives for involvement:  *‘It was like that psychological aspect of it really. Because we rarely touch on that … (and) I thought it’d be a really good skill to have … be good to explore.’* (Jamie)  *‘It was a new thing, and … (because) the amputation rate in (City) is quite high … I was quite interested in getting more knowledge, more expertise in that kind of area.’* (Grace)  *‘(It’s) a great opportunity … it gives you something, a remote position, without moving … an opportunity to work in a different way, a fulfilling way … (and) a new skill.*’ (Marilyn) |
| **Training and other learning** | ***Training: a lot of new learning***  Overall experience:  *‘I loved, I really enjoyed the training. It was, it was brilliant … (but) even though I know about … CBT … there was a lot of information … a lot to take in, in a short amount of time.’* (Regina)  *‘The training was really good … it was long (but) it needed to be … I had a bit of a background in some of the stuff, but I still learnt loads of new stuff.’* (Susan)  Key learning:  *‘I definitely did feel like I learnt … a bit more about foot health from the training.’* (Martine)  *‘The learning that I took away (from training) was … that it’s not about telling people what they need to do, it’s helping them work out what their goals are, and how they can make small changes. And working in a collaboration … together … focusing on what the client wants to change.’* (Regina)  Learning from peers:  *‘There was six of us … three of us were podiatrists … two were from a psychology background and one … (from) some other health profession (…) The very fact that we were a diverse group, I found very helpful, because it helped me look at the training in multiple ways, not just … with a podiatry hat on (…) You learn from each other … (and) you can assist each other … having a multi-professional group of people enriches the conversation.’* (Frederica)  *‘I was (training) with another girl … and she knew the techniques, but she didn’t know anything about feet. So, we were kind of like opposites … It was good to see her at work, as well as (Trainer) (…) (We) would quite often practice together, and I learnt a lot from her. And I think she did from me too.’* (Marilyn) |
|  | ***Independent learning: addressing gaps in, or attrition of, knowledge***  Perceiving gaps in their own knowledge and/or skills  *‘I’m … not like a diabetes healthcare professional. And … that made me worry a little bit … even though I know it’s not about giving diabetes advice.’* (Regina)  Wanting to do their best:  *‘I just really wanted to … do my best … for the trial … (I) just wanted to do a good job.’* (Susan)  *‘It is just the way I approach everything. I, I want to do things well. And I will put in a lot of extra work, into everything I do, to ensure that I do it well.’* (Georgina)  Taking steps to enhance knowledge:  *‘Later on in the course, I didn't feel as though I was so well prepared. I really had to re-read (the materials).’* (Cath)  *‘Training was in November, (but) I didn’t start until June. And by that time, well, it had all gone out of the window. So I had to do it all again … by just going through the programme – the maintenance booklet, the participant booklet, and the facilitator handbook … and I watched the videos and … looked at the PowerPoints again.’* (Frederica)  *‘I said to my colleague … if this was an ongoing thing, I would invest a lot more time now … I’m sort of through the initial … induction … trial period, and now I need to really strengthen some of my areas, more academically … go back to the online materials … review it all.’* (Susan) |
|  | ***Experiential learning: application, familiarity, and (appropriate) challenge***  Having opportunities to apply learning  *‘Was I ready? I would say, yes, I was ready to do it … I was nervous … (but) I had to put my big girl’s pants on, and just do it … (Because) for me, unless I do it, I’m not gonna learn.’* (Josie)  *‘You needed to actually put it into practice, quite quickly, I think, after you’d done the training, in order to build your confidence in it.’* (Marilyn)  (Or not)  *‘I only managed to work with two patients … (and) the first patient dropped out after a couple of sessions.’* (Martine)  *‘I wouldn’t … say I’m even remotely experienced at delivering this. I still feel very novice.’* (Susan)  Becoming familiar with the intervention  *‘I did, very late in the day, suddenly realise that I needed to talk about (the maintenance website) with him, and at that point I had no idea how … (With others) I was a bit more prepared, so I started to talk about it (earlier) …asking them to access it, and have a look.’* (Edwina)  *‘I had the experience … (and) I knew the handbook really well. And that certainly helped me with (participant with unique needs).’* (Charlie)  *‘At the beginning, I found it very challenging ... Then, over time, I think you develop that confidence, and the expertise … (to) apply that level of curiosity, and just ask them to expand on … X, Y and Z …(So) I think as I did more patients, I became more confident doing the CBT with them.’* (Janice)  Experiencing (appropriate) challenge  *‘I were petrified, absolutely petrified to do the first week … (but) he was such a nice … chappie … (and) put me so much at ease.’* (Winnie)  *‘I think the first chap, certainly, would have been very, very challenging to do (as a new facilitator), because I think having had the confidence to say, I’ve just got to trust the process, and keep going, and this will come good in the end (was key).’* (Charlie) |
|  | ***Reflective practice: transforming challenges into learning opportunities***  Self-awareness, review and reflection  *‘You’re tempted to actually guide them, advise them, but I had to hold myself back.’* (Grace)  *‘To start with, I would listen to those sessions in full, myself, however excruciating it was. And … I did learn from them. And … they continued to be very useful, bits that I wanted to check. I still regularly listen to, to bits.’* (Georgina)  *‘Realising what worked, and what didn’t work, helps you plan how to tackle it next time.’* (Frederica)  Recognising where struggling  *‘One, it was like pulling teeth. I struggled the whole way to engage them, to get them to even have a conversation with me. You’d ask … an open-ended question and they’d give you about five words as an answer … I found that particular person really, really difficult.’* (Frederica)  *‘I had one that withdrew, and it … was quite difficult … you do take it personally.’* (Cath)  *‘I had a point when I didn’t have anyone who was engaging very well … Then you start thinking, well, maybe that’s me, maybe I’m not doing it well enough.’* (Georgina)  Reaching out for support  *‘Not all the time are things gonna go well. And that’s what you take into supervision.’* (Regina)  *‘It was very useful to check something out with (Name). I liked … that safety of going, actually, can you re-, can you listen to that? Have I missed something? Is there something else I could have done?’* (Josie)  *‘With any of the challenging ones, I’ve always kind of lifted them up, to (Supervisor) and asked for guidance.’* (Janice)  *‘I had conversations with (Supervisor) about the CBT aspect of it … I was struggling with that more than I anticipated … my first patient … just didn’t get it at all … and I just felt at a loss as to how to proceed.’* (Edwina)  Learning with and from peers  *‘I found it really helpful not only to like bring my patient to the supervision, and sort of discuss (him/her) as a group, I also found it really helpful to like listen to other … professionals’ like experiences as well, ‘cause often there was a lot of like things that they were discussing that I could apply to my sessions too. And also, just some reassurance, ‘cause I think it was unfortunate that the first person … dropped out after the first session. And I went back to supervision, and I was like, oh my God, is it something I’ve done? Like, you know, why has this person dropped out? And then to hear from other people … that does happen, it’s happened to me as well … was just nice – to get that reassurance that it’s not something that you’re doing. So yeah, I think it was good in terms of like learning from others, but also getting that like reassurance from the group as well.’* (Martine)  *‘It was good to hear other people’s scenarios that they were bringing to the sessions, because it just added to your knowledge of what might come up. And that was, that was good learning. And it’s also nice to just hear other people’s issues. (*laughs*) You don’t feel … quite so alone, just thinking, why am I, you know, why is this not quite working?’* (Charlie)  *‘Working … (from) home … it’s quite isolating … (so) it was lovely being able to link into the (group) supervision sessions – to bring any challenging patients and just to kind of listen to others’ (ideas) … because we’re all different … the facilitators are from different backgrounds, so it was good to share best, best practice I guess, (and) support for each other.’*  (Janice) |
| **Returns and rewards** | Satisfaction/enjoyment  *‘You know, it did feel like an achievement at the end of it. My goodness, I’ve managed to do Zooms, and meetings, and MS Teams, and audio-record, and follow a plan. There was a lot of, a sense of achievement of stuff out of my comfort zone, at the end of it.’* (Charlie)  *‘It’s just been a real privilege, for me, to be involved with REDUCE … it’s just been a real pleasure ... I’d look forward to meeting new people, … working with them, and learning more, and … I hope the participants have benefited even half as much as I have, because it’s been just great for me.’* (Georgina)  *‘I love(d) the work, I really enjoy(ed) speaking with … participants … I found that really rewarding. I feel a bit sad that it’s now finished for me … I enjoyed the work, I got paid, I met (some) fabulous people, who I will always remember, because they did touch me … Hopefully I touched (them) as well, through this programme. And so I’m glad I’ve been a part of it.’* (Frederica)  Impact on routine practice  *‘How it’s changed my practice? I now take more time to discuss feet … not just a superficial (enquiry) … I actually have a conversation with (patients) about foot care … about how they can manage it themselves, practically, because most of the patients that come, they always think that management is when you sit in podiatry, so they don’t do anything else for their feet.’* (Grace)  *‘I think you listen more … to what your patients are saying (…) I think it’s … enhanced my confidence as a … listening podiatrist.’* (Janice)  *‘Just listen(ing) to people (…) it’s a real eye-opener … I have so much more empathy for people in that position (now) (…) and (how) doing … things that clinically sound quite simple … (with) everything in their lives that they’re trying to juggle… It’s something else.’* (Charlie)  *‘When (I’m) giving patients advice about, their health, (I’m) also finding out what their views (are) on the advice I’ve given them – whether they think it’s practical, or whether they think there’s anything that’s going to get in the way.’* (Alica)  Wider benefits  *‘I’ve also learnt so much from that programme.’* (Grace)  *‘What I think it has done is it’s allowed me to explore how, if, if people, if my family, for instance, are struggling with something, then instead of me jumping in with two feet and saying, well, you need to do this that and the other, I now say, well how does that make you feel? And ... what will you do about it? And … if you did X, Y, Z, what will change, will happen? And so I think … it’s helped me, counsel … family, and friends.’* (Frederica) |
